# The physician and hereditary angioedema friend or foe: 62-year diagnostic delay and iatrogenic procedures

**DOI:** 10.1186/s13223-018-0275-4

**Published:** 2018-10-25

**Authors:** Anna Valerieva, Marco Cicardi, James Baraniuk, Maria Staevska

**Affiliations:** 1 Clinical Center of Allergology, Clinic of Allergy and Asthma, University Hospital “Alexandrovska”, Medical University of Sofia, 1, Georgi Sofiiski Str., 1431 Sofia, Bulgaria; 2 Department of Biomedical and Clinical Sciences, ASST Fatebenefratelli Sacco, Luigi Sacco Hospital-Polo Ospedaliero, University of Milan, Milan, Italy; 3 Division of Rheumatology, Immunology and Allergy, Department of Medicine, Georgetown University, Washington, DC USA

**Keywords:** Hereditary angioedema, Diagnosis delay, Misdiagnosis, C1-inhibitor deficiency, C1-inhibitor esterase, Iatrogenic procedure, Recurrent ascites, Recurrent ileus

## Abstract

**Background:**

Hereditary angioedema due to C1 inhibitor deficiency (C1-INH-HAE) is a rare autosomal dominant disease characterized by episodes of acute subcutaneous swelling, and/or recurrent severe abdominal pain. The disease is potentially fatal if the upper-airway is involved. Iatrogenic harm can occur if HAE is not considered in the differential diagnosis, the specialists are not aware of the natural history, diagnosis and treatment of HAE, or as a result of unnecessary surgical and other iatrogenic interventions.

**Case presentation:**

We present the case of a 72-year-old man who began suffering recurrent abdominal pain at the age of 8 years. The pain led to frequent emergency department visits, three emergency surgical interventions, and 5 endoscopies before C1-INH-HAE was diagnosed at the age of 70. Infrequent subcutaneous swellings were attributed to unknown allergic reactions that were not related to the primary diagnosis of abdominal pain. Family history was positive for recurrent abdominal pain and angioedema but was ignored until the propositus’ grandson developed recurrent severe oro-facial edema attacks. The boy’s mother searched the worldwide web and found educational materials on a patient association website. She suggested complement C4 and C1-INH testing that led to the appropriate diagnosis of C1-INH-HAE type 1 in her son and his grandfather.

**Conclusion:**

This report emphasizes the importance of accurately evaluating personal and family history in patients with a long history of recurrent, acute, severe but medically unexplained abdominal pain and cutaneous swellings. Here, the diagnosis of HAE was overlooked for 62 years and the focus on abdominal complaints led to numerous surgical interventions without consideration of the full differential diagnosis. Screening family members from all generations for unrecognized angioedema, abdominal pain, and measurement of C1-INH and C4 are essential for accurate and timely diagnosis of HAE.

## Background

Hereditary angioedema (HAE) is a rare autosomal dominant disease characterized by recurrent swellings of the extremities, oro-facial-pharyngeal zones, upper airways, and genitalia [1]. These lead to frequent emergency department (ER) visits. More than 90% of subjects with cutaneous angioedema episodes also develop recurrent edema of the intestinal mucosa causing severe abdominal pain [2, 3]. The most common and best-characterized form of HAE is due to mutations of the C1 inhibitor (C1-INH) gene (*SERPING1*) resulting in reduced (below 50% of normal) C1-INH plasma levels (C1-INH-HAE type 1) and/or function (C1-INH-HAE type 2). Marked consumption of complement C4 to below 10 mg/100 mL supports the diagnosis. C4 measurement is a good screening test that is performed by most clinical laboratories.

Hereditary angioedema is a potentially life threatening disease, with 1–3% of all angioedema episodes affecting the larynx and upper airways. In the absence of appropriate diagnosis and treatment, 25% of patients die from asphyxiation [4–6]. The burden of HAE has a huge geographical discrepancy due to differences in treatment availability. Beginning in 2009 new drugs for C1-INH-HAE were approved in western Europe and North America. Treatment availability has decreased mortality to near zero if the correct diagnosis is known. Therefore, it is essential to increase awareness of how to diagnose and treat HAE and to reduce any delays in diagnosis. Nevertheless, extreme delay is still a major issue, even in developed countries with good health care systems [7–9].

In countries where new generation treatments are not available, awareness of the disease is minimal, patient lives are disrupted, and mortality for HAE remains high. We highlight this situation by reporting a patient with a 62-year history of typical subcutaneous and abdominal symptoms who had numerous gastrointestinal interventions, and whose diagnosis was suggested by a family member who noted the positive family history.

## Case presentation

**Fig. 1:**
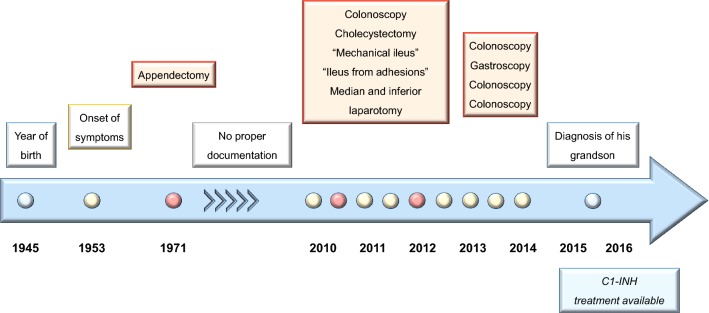
Timeline

A 72-year old man suffered from recurrent abdominal attacks beginning at age 8 (Fig. 1, Timeline). The attacks usually began as a colic-like periumbilical pain that spread to involve the entire abdomen, mimicking abdominal guarding, and progressing with vomiting, diarrhea, debilitation, and often syncope. During adolescence, infrequent subcutaneous angioedema episodes occurred and could not be related to any specific cause. Sometimes, an ill-defined, non-pruritic skin “rash” occurred in various locations. There was no evidence of any atopic condition such as allergic rhino-conjunctivitis, atopic dermatitis or asthma, and no specific tests for allergy were performed. Family history was positive as his father had similar symptoms; however, this evidence went ignored by the consulting physicians. Later, our patient started to experience peripheral subcutaneous swellings, often as a result of minor traumatic stimuli. Conventional anti-allergic therapy with antihistamines, with or without corticosteroids, had scarce benefit. No matter the treatment, these attacks lasted for 48–72 h, or more. He had frequent ER visits over his entire lifetime.

In 1971, at the age of 26, he underwent surgery with a diagnosis of catarrhal appendicitis. There is a limited clinical documentation for the next 30 years, during which he experienced his stereotypical, recurrent abdominal episodes and had 4–6 hospital admissions per year with no conclusive diagnosis.

Recurrent abdominal symptoms in February 2010, led to emergency colonoscopy with removal of a benign polyp. No other diagnosis was made.

One month later, in March 2010, the recurrent acute abdominal pain was diagnosed as acalculous cholecystitis. Ultrasound examination showed ascites in all abdominal compartments, and radiography showed hydro-aeric shadows in the intestinal region. Laparoscopic cholecystectomy was performed without objective evidence for gallbladder lithiasis. Diagnosis at discharge was catarrhal cholecystitis and laparoscopic cholecystectomy.

Two weeks later the patient was hospitalized for severe abdominal pain interpreted as mechanical ileus. Ascites and hydro-aeric shadows were present. Computed tomography (CT) showed 13 mm thickening of the jejunal wall. Symptoms resolved after 7 days of conservative spasmolytic and analgesic treatment.

Seven weeks later in June 2010 he was hospitalized with the identical clinical picture and given the diagnosis of “recurrent mechanical ileus.” An “urticarial rash” was documented on admission. Physical examination found extensive abdominal tympanism, with radiographic hydro-aeric shadows in the left side of the abdomen. Symptoms resolved after 5 days of conservative therapy.

In October 2010 he presented with analogous symptoms. Diagnosis this time was presumed to be an acute volvulus and he had an emergency median and inferior laparotomy. The procedure was described as “manual fixation of a volvulus without intestinal resection, plus debridement of peritoneal adhesions.” The post-operative period was complicated by severe abdominal pain that was resistant to combined analgesic therapy with opioids and nonsteroidal drugs (NSAIDs).

In 2011 and 2012, the patient’s recurrent abdominal pain attacks lead to 4 emergency colonoscopies and 1 gastroscopy. Several benign polyps were extirpated, but there was no indication they were related to the symptoms. Between 2013 and 2015, repeated emergency visits resulted in hospital admissions to Departments of Internal Medicine, Gastroenterology, and Intensive Care, with no conclusive diagnosis at discharge. Additional surgery and endoscopies were proposed, but were refused by the patient based on his previous experiences of multiple ineffective, and in fact, worsening his suffering interventions.

**Fig. 2:**
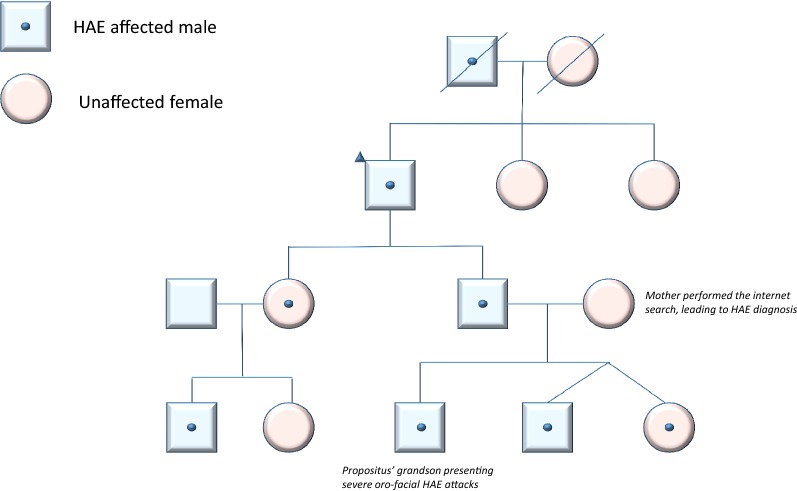
Family tree

In 2015 one of his grandsons developed episodes of recurrent, disfiguring subcutaneous oro-facial angioedema, abdominal pain and swellings of the oro-pharyngeal mucosa (lips, epi- and hypopharynx, and uvula) that lasted up to 1 week, and were totally resistant to conventional anti-allergic therapy with epinephrine, corticosteroids and antihistamines. No association with an allergic condition could be made: no food, drug, or other triggers precipitated the episodes. They seemed to be unpredictable, or in some occasions provoked by traumatic stimuli. The child had frequent emergency visits and hospitalizations of 3–7 days’ duration. His mother noticed that similar symptoms of facial swellings and abdominal pain occurred in at least three generations of her husband’s family. She searched the internet for these symptoms and began to suspect hereditary angioedema. She requested that her son have C4 and C3 measured. The results showed low C4 and normal C3, so the presumptive diagnosis of HAE was made and the boy was referred to our tertiary center. Further evaluation confirmed the diagnosis of C1-INH-HAE type I in the child and 6 other family members (Fig. 2, Family tree). Specific treatment for bradykinin-mediated angioedema, C1-INH concentrate on demand: recombinant in the adults (Ruconest^®^, conestat alfa), and plasma-derived in the children (Berinert^®^) was introduced in all family members with good clinical outcomes.

## Discussion

C1-INH-HAE has a clear-cut clinical presentation of recurrent cutaneous swellings, abdominal pain and pharyngeal/laryngeal edema. Erythema marginatum is a typical manifestation that often precedes the angioedema, and could be easily mistaken for urticaria by non-specialists, although non-pruritic. The lips, face and pharynx become severely edematous as the loose connective tissue fills with extravasated plasma due to the localized vascular leakage. Unlike histaminergic angioedema (as in urticaria or other allergic conditions), the edema persists for several days.

Angioedema of the serosa and walls of the intestines leads to thickening of the visceral walls, compression of the lumen, and clinical picture of temporary ileus leading to emesis and diarrhea. Serosal extravasation leads to ascites that is recurrent and spontaneously resolving. The combination of these oro-facial, peripheral cutaneous and gastrointestinal manifestations places HAE firmly in the routine differential diagnosis of recurrent unexplained abdominal pain [10]. Furthermore, the positive family history increases the probability for this otherwise rare condition, as the disease segregates in family members with the congenital plasma deficiency of C1-INH and subsequent consumption of C4.

The skin manifestations recognized as prodromes of angioedema could contribute to the difficulty of diagnosing HAE. Erythema marginatum is a typical manifestation that precedes the occurrence of angioedema attacks in C1-INH-HAE. It can be easily mistaken for hives and lead to the misdiagnosis of allergy or chronic urticaria [11]. In urticaria degranulation of mast cells by IgE (atopy), autoantibodies, and other agents such as nonsteroidal anti-inflammatory drugs and intravenous contrast dyes lead to release of histamine in the superficial dermis and mucosal lamina propria. Histamine causes an intense pruritus with localized vascular leak, but the duration of the urticaria is generally less than 24 h. Mast cell disorders respond well to antihistamines, corticosteroids, and, in an, emergency, epinephrine. C1-INH-HAE is not a mast cell mediated disorder, as these medications are ineffective, as seen in our patient. In HAE trauma, infection and other unknown triggers lead to activation of plasma kallikrein by factor XIIa and subsequent massive release of bradykinin from cleaved high molecular weight kininogen when their controller, C1-INH, is deficient [12]. Bradykinin leads to a painful sensation, and causes deep dermal and mucosal vasodilation, vascular leak, and plasma extravasation. This is supported by the efficacy of icatibant, a bradykinin B2 receptor antagonist, in C1-INH-HAE [13]. The histological difference between histamine H1 and bradykinin B2 receptor bearing neurons and vessels in the upper versus lower dermis, respectively, contributes to the clinical and etiological distinctions between histaminergic and bradykinin-mediated angioedema. This difference has an important implication as it explains the uselessness of antihistaminics and other treatments targeted to mast cells versus bradykinin antagonist and C1-INH concentrate. C1-INH (plasma-derived and recombinant forms) and bradykinin antagonists are now the standard of care for acute attacks of C1-INH-HAE [14]. Failure to use these drugs exposes HAE patients to the risk of death and extensive suffering. Several bradykinin-targeted drugs have become available in the last 10 years and new ones are moving through the development pipeline. Unfortunately, all these new therapies are extremely expensive and have limited or no availability in low-income countries [15]. In Bulgaria, the country of our patient, recombinant C1-INH became available for HAE treatment in 2013 and still has limited availability and strictly controlled access for patients diagnosed with C1-INH-HAE Type 1 and 2. The limited therapeutic options for angioedema may reduce the motivation to reach a diagnosis for both patients and physicians [16], and provides an additional obstacle to reach correct diagnosis.

Our patient developed a complete presentation of HAE at the age of 8, but was not diagnosed for over 60 years. This was despite over countless emergency department and hospital admissions, innumerable clinical evaluations by many teams of physicians over the decades, invasive surgical and endoscopic interventions. Our patient confirms that physicians may still ignore the medical dogma of carefully investigating family history. His delay in diagnosis is not unusual, as the average time to diagnosis of HAE is between 12.1 and 12.9 years [17, 18]. The delay may be longer if abdominal symptoms predominant the clinical presentation. Undiagnosed patients have up to nine times higher risk of asphyxiation [5] and an overall risk of death from laryngeal edema of 25%. The rate of iatrogenic harm due to unnecessary surgical procedures in diagnosed and undiagnosed patients is not known, though may be substantial [19]. This is another reason to understand the pathophysiology of HAE, aim to accelerate the time to diagnosis, and provide life-saving modern therapies.

Inheritance of C1-INH-HAE is autosomal dominant, but only 75% have an affected family member. Diagnosis is confirmed by plasma deficiency of C1-INH protein level and function. The remaining 25% of index cases are sporadic and due to de novo mutations in *SERPING1* [20]. Because these patients have no affected ancestors, their family history will be negative and it may be more difficult to reach the diagnosis. However, even patients with positive family history can have delayed diagnosis, as for example in an Iranian population where those with positive family history have a longer delay compared to patients with no such anamnesis [21].

The differential diagnosis of HAE includes acquired C1-INH deficiency. It has a similar clinical presentation but presents at older ages [22]. Autoantibodies to C1-INH may be responsible and occur as a paraneoplastic syndrome. The acquired form can have low C1q levels, while these remain normal in hereditary angioedema [23].

An integral part of this case was the path to discovery of the diagnosis. The unaffected mother of a sick child performed the internet search that suggested the correct diagnosis. This highlights the importance of accurate, publically available information provided by patient associations (http://www.haei.org) and the general paradigm shift towards patient-centric, individualized diagnosis and treatment in rare diseases. Educational programs for patients increase the probability that they and their relatives become knowledgeable about the disease. These programs and social media become tools to integrate medical education directly to patients [24, 25]. In addition, providing up-to-date medical information for emergency physicians, gastroenterologists, internists, family doctors, allergists and others who see angioedema patients is essential and should be widely encouraged, as such common efforts could help prevent further HAE misdiagnosis, delay of adequate medical care, iatrogenic procedures, and complications.

Special attention must be dedicated to address the familial inheritance of the condition, as HAE affects the lives of many consecutive generations. Therefore, wholesale screening by history and physical, with targeted C1-INH and C4 testing should become the standard of practice for all family members of a patient with HAE.

## Abbreviations

HAE: hereditary angioedema
C1-INH: complement C1 inhibitor
C1-INH-HAE: hereditary angioedema due to C1-inhibitor deficiency
